# The RNA demethylase FTO promotes glutamine metabolism in clear cell renal cell carcinoma through the regulation of SLC1A5

**DOI:** 10.1126/sciadv.adv2417

**Published:** 2025-06-18

**Authors:** Man Zhao, Dalin Zhang, Haowen Jiang, Nishanth Kuganesan, Suchitra Natarajan, Leighton Pu, Kaushik N. Thakkar, Hongjuan Zhao, Quynh Thu Le, Amato J. Giaccia, James D. Brooks, Donna M. Peehl, Jiangbin Ye, Erinn B. Rankin

**Affiliations:** ^1^ Department of Radiation Oncology, Stanford University, Stanford, CA 94305, USA.; ^2^ Department of Urology, Stanford University, Stanford, CA 94305, USA.; ^3^ Department of Oncology, University of Oxford, Oxford, UK.; ^4^ Department of Radiology and Biomedical Imaging, University of California, San Francisco, CA 94158, USA.; ^5^ Department of Obstetrics and Gynecology, Stanford University, Stanford, CA 94305, USA.

## Abstract

Glutamine reprogramming plays a crucial role in the growth and survival of clear cell renal cell carcinoma (ccRCC), although the mechanisms governing its regulation are still not fully understood. We demonstrate that the RNA demethylase fat mass and obesity-associated gene (FTO) drives glutamine reprogramming to support ccRCC growth and survival. Genetic and pharmacologic inhibition of FTO in ccRCC cells impaired glutamine-derived reductive carboxylation, depleted pyrimidines, and increased reactive oxygen species. This led to increased DNA damage and reduced survival, which could be rescued by pyrimidine nucleobases or the antioxidant *N*-acetylcysteine. Mechanistically, FTO demethylates the glutamine transporter solute carrier family 1 member 5 (SLC1A5) messenger RNA to promote its expression. Restoration of SLC1A5 expression in FTO-knockdown cells rescued metabolic and survival defects. FTO inhibition reduced ccRCC tumor xenograft and PDX growth under the renal capsule. Our findings indicate that FTO is an epitranscriptomic regulator of ccRCC glutamine reprogramming and highlight the therapeutic potential of targeting FTO for the treatment of ccRCC.

## Teaser

FTO is an epitranscriptomic regulator of glutamine reprogramming in kidney cancer.

## INTRODUCTION

Renal cell carcinoma (RCC) is a major cause of patient morbidity and mortality with approximately 400,000 cases and 175,000 deaths per year (*1*). The most common and aggressive subtype of kidney cancer is clear cell renal cell carcinoma (ccRCC), which accounts for 70% of all kidney cancer cases (*2*). While surgery is the standard care for patients with localized disease, approximately 30% of patients with ccRCC are diagnosed with regionally advanced or metastatic disease and require systemic therapy (*2*–*4*). Despite current antiangiogenic agents and immunotherapies that target the tumor microenvironment, the 5-year survival rate for patients with ccRCC with advanced disease remains only 14% (*5*). Hypoxia-inducible factor 2 alpha (HIF-2) plays an important oncogenic role in ccRCC, which has led to the recent development of small-molecule HIF-2 inhibitors. The HIF-2 inhibitor belzutifan was approved by the Food and Drug Administration (FDA) in 2021 for the treatment of ccRCC associated with von Hippel Lindau (VHL) disease and in 2023 for advanced RCC (*6*, *7*). However, only 49% of patients with VHL disease–associated ccRCC and 25% of patients with advanced sporadic ccRCC met objective response rates with belzutifan treatment (*8*–*12*). These findings highlight the unmet clinical need for additional therapies that can directly inhibit the growth and survival of ccRCC cancer cells.

Metabolic reprogramming is a hallmark feature of ccRCC. VHL-deficient ccRCC tumors are dependent on glutamine metabolism for growth and survival. Functionally, ccRCC tumors take up exogenous glutamine and use glutamine as an important carbon and nitrogen source (*13*). Glutamine is converted into glutamate and α-ketoglutarate and metabolized through reductive carboxylation to produce citrate and lipids (*14*). In addition, glutamine is used to generate aspartate for pyrimidine biosynthesis and glutathione for redox balance as well as for the production of asparagine and nucleotide metabolism intermediates (*13*, *15*). Targeting glutamine reprogramming for the treatment of ccRCC is an attractive strategy, as glutamine utilization is increased in ccRCC compared to normal kidney tissues (*16*, *17*) and up-regulation of glutamine pathways is associated with high grade, high stage, and metastasis in ccRCC (*16*). However, the mechanisms driving glutamine reprogramming in ccRCC remain poorly understood.

The RNA demethylase fat mass and obesity-associated gene (FTO) is emerging as an important oncogenic factor in ccRCC. N6-methyladenosine (m^6^A) modifications on mRNA adenosine residues are the most common internal mRNA modification found transcriptome-wide in at least 25% of all RNAs. They are reversible and dynamic with writer, reader, and eraser proteins that regulate gene expression and biological processes similar to DNA and histone modifications (*18*). FTO belongs to the AlkB family of Fe (II)– and 2-oxoglutarate–dependent N^6^-methyladenosine RNA demethylases that regulate pre-mRNA splicing, mRNA translation, degradation, and nuclear export (*19*, *20*). Functionally, FTO has been proposed to promote cancer cell growth and survival through the regulation of oncogenes (*21*), antiapoptotic factors (*22*), immunosuppressive factors (*23*), and glycolysis (*24*, *25*). However, there is limited research on its role in glutamine reprogramming.

We recently found a synthetic lethal role for FTO in VHL-deficient ccRCC (*26*). Notably, FTO knockdown reduced VHL-deficient growth and survival independent of HIF-2 (*26*). Recent work confirmed that FTO knockdown reduces the growth of ccRCC in a HIF-2–independent manner (*27*, *28*). Yet, actionable mechanistic insights into how FTO promotes ccRCC growth and survival and the therapeutic potential of FTO-based therapy in ccRCC remain poorly understood.

Here, we demonstrate a role for FTO in the regulation of glutamine uptake and metabolic reprogramming in ccRCC through the regulation of solute carrier family 1 member 5 (SLC1A5). We show that FTO drives glutamine reprogramming in ccRCC cells, supporting growth and survival. Inhibition of FTO impaired glutamine-derived reductive carboxylation, depleted pyrimidines, and increased reactive oxygen species, leading to DNA damage and reduced survival, which could be rescued by pyrimidines or antioxidants. FTO demethylates SLC1A5 mRNA to promote its expression and restoring SLC1A5 in FTO-knockdown cells rescued metabolic and growth defects. FTO inhibition reduced ccRCC tumor growth, suggesting FTO as a potential therapeutic target for ccRCC.

## RESULTS

### FTO inhibition reduces glutamine-derived eductive carboxylation and de novo pyrimidine synthesis in ccRCC cells

**Fig. 1.**
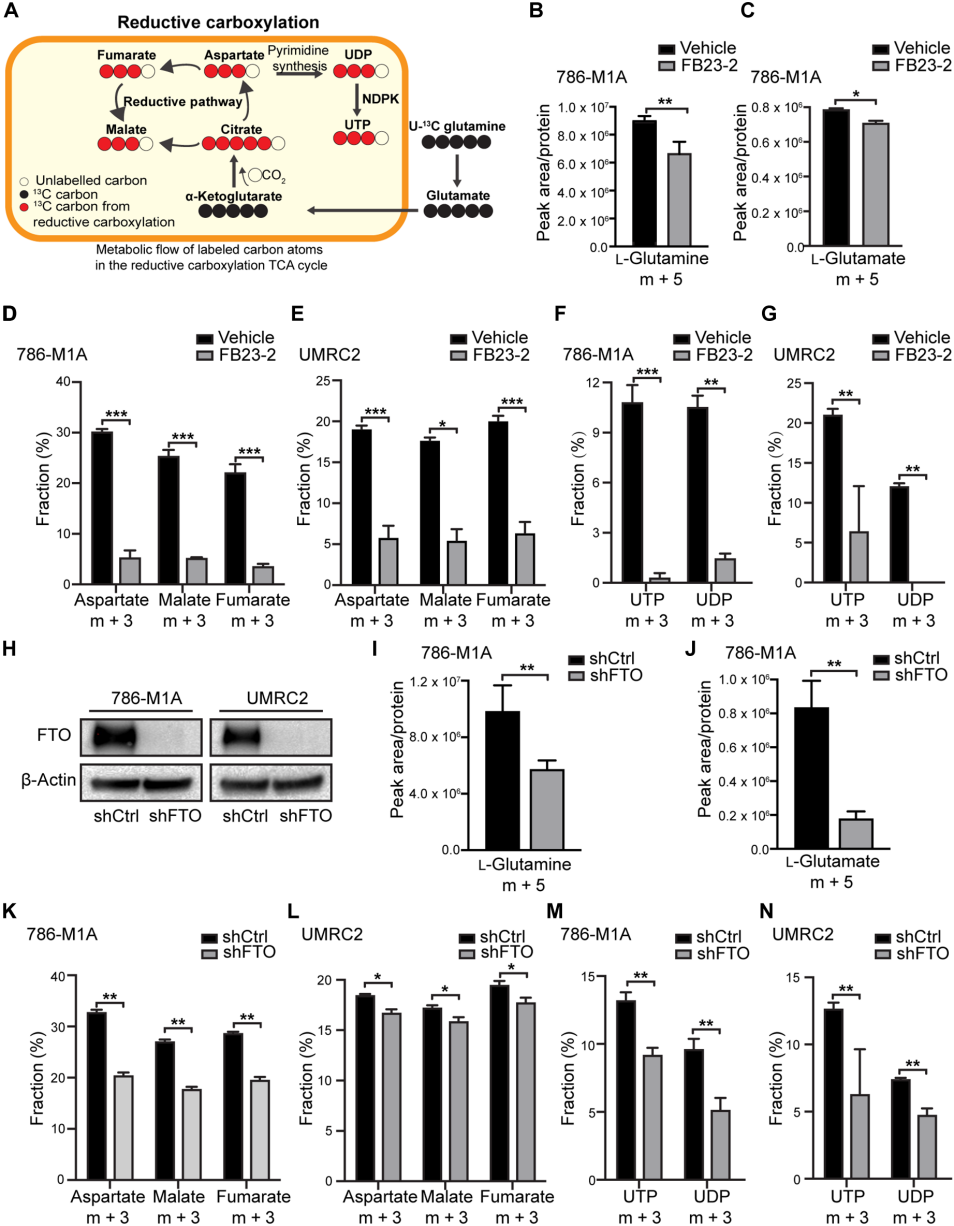
FTO inhibition reduces glutamine-derived reductive carboxylation and de novo pyrimidine synthesis in ccRCC cells. (**A**) Schematic of U-^13^C glutamine tracing studies. Diagram of the metabolic flow of labeled carbon atoms in the TCA cycle of reductive carboxylation. Red dots denote ^13^C carbon atoms originating from uniformly labeled U-^13^C glutamine. (**B** and **C**) 786-M1A cells were pretreated with DMSO or 5 μM FB23-2 for 72 hours, then labeled with U-^13^C glutamine in complete medium for 10 min. Total levels of m + 5 l-glutamine and l-glutamate were measured using LC-MS and normalized to total protein levels. (**D** to **G**) 786-M1A and UMRC2 cells were labeled with U-^13^C glutamine for 2 hours after pretreatment with DMSO or 5 μM FB23-2 for 72 hours. (D and E) The fraction of m + 3 aspartate, malate and fumarate were analyzed using LC-MS. (F and G) The fraction of UTP and UDP was measured using LC-MS. (**H**) Doxycycline (Dox)–inducible shFTO knockdown was verified by Western blot analysis in 786-M1A and UMRC2 cells at 5 days post-Dox (2 μg/ml) treatment. (**I** and **J**) 786-M1A shCtrl and shFTO cells were treated with vehicle or Dox (2 μg/ml) for 5 days, then labeled with U-^13^C glutamine for 10 min. The metabolite enrichment of m + 5 l-glutamine and l-glutamate was measured by LC-MS. (**K** to **N**) 786-M1A and UMRC2 shCtrl and shFTO cells were pretreated with Dox (2 μg/ml) for 5 days, then labeled with U-^13^C glutamine for 2 hours. (K and L) The fractions of m + 3 aspartate, malate, and fumarate were analyzed using LC-MS. (M and N) The fractions of UTP and UDP were measured using LC-MS. Each column represents the mean ± SD. Biological replicates (*n* = 3) were analyzed in each group. Statistically significant differences are indicated: *P < 0.05; **P < 0.01; ***P < 0.001; determined by the Student’s two-tailed *t* test.

Previous studies have established that VHL-deficient ccRCC tumors are dependent on glutamine metabolism for growth and survival (*13*–*15*). Our previous work identified the glutamine transporter SLC1A5 as an FTO target in ccRCC cells (*26*). However, the functional role for FTO in the regulation of glutamine reprogramming in cancer cells remains unknown. We first confirmed that the VHL-deficient ccRCC cell lines used in this study (UMRC2 and 786-M1A), require glutamine for growth and survival (fig. S1, A to D). To determine whether FTO inhibition influences glutamine uptake and/or downstream reductive carboxylation in ccRCC cells, we performed U-^13^C glutamine tracing studies. U-^13^C glutamine labels glutamate and α-ketoglutarate (α-KG) as m + 5. When α-KG undergoes oxidation, it results in m + 4 labeling in other TCA cycle intermediates, while reductive carboxylation produces m + 5 citrate and m + 3 in metabolites including malate, aspartate, and fumarate [Fig. 1A, (*29*)]. Pretreatment for 72 hours with a potent and selective FTO inhibitor, FB23-2 (*30*), reduced glutamine uptake and conversion to m + 5 glutamate in ccRCC cells (Fig. 1, B and C). In addition, U-^13^C glutamine tracing revealed that the FTO inhibitor FB23-2 inhibited reductive carboxylation in ccRCC cells with reduced fraction of the reductive metabolites m + 3 aspartate, malate, and fumarate and reduced m + 3/m + 4 aspartate, malate, and fumarate ratios (Fig. 1, D and E, and fig. S1, E and F). As aspartate is used in the de novo synthesis of pyrimidines, we also observed that FB23-2 decreased glutamine-derived m + 3 UTP and m + 3 UDP in ccRCC cells (Fig. 1, F and G).

Next, we determined whether genetic knockdown of FTO has a similar effect on ccRCC glutamine uptake and downstream metabolism. Similar to the pharmacologic inhibition of FTO, FTO knockdown reduced glutamine uptake, conversion to glutamate, downstream reductive carboxylation, and pyrimidine synthesis in ccRCC cells (Fig. 1, H to N, and fig. S1, G and H). Collectively, our data indicate that both pharmacologic and genetic FTO inhibition reduces glutamine uptake and reductive carboxylation in ccRCC cells.

### FTO inhibition reduces GSH synthesis and increases intracellular ROS levels in ccRCC cells

**Fig. 2.**
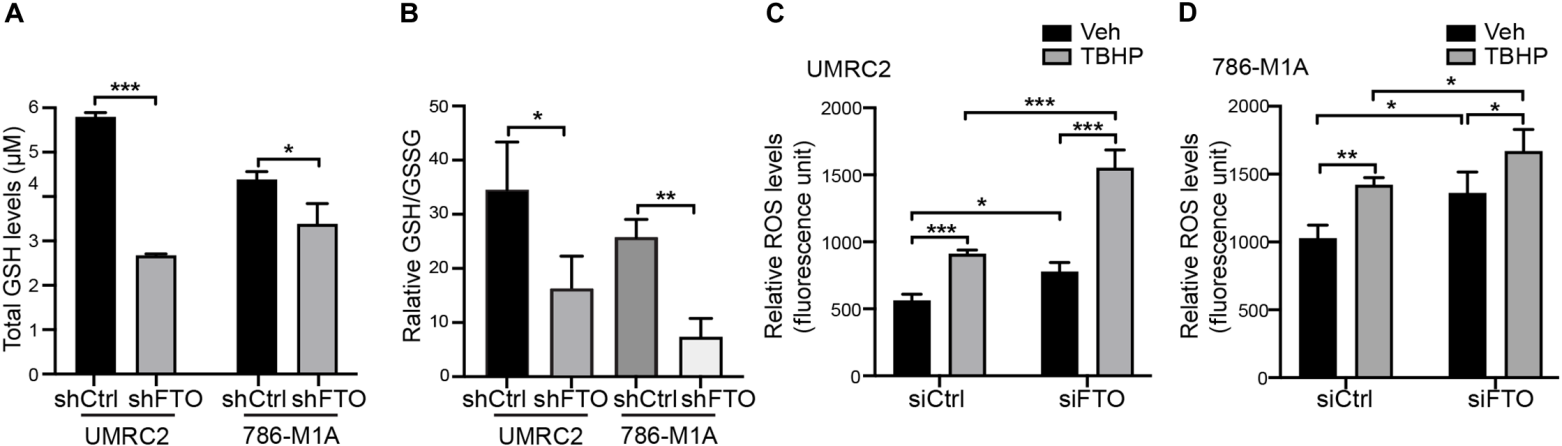
FTO inhibition reduces GSH biosynthesis and increases intracellular ROS levels in ccRCC cells. (**A**) Total GSH levels in shCtrl and shFTO UMRC2 and 786-M1A Dox-inducible cell lines treated with Dox for 72 hours. (**B**) Relative GSH/GSSG ratio in UMRC2 and 786-M1A Dox-inducible shCtrl and shFTO cell lines. (**C**) Intracellular ROS levels were determined by carboxy-DCFDA staining in UMRC2 cells transfected with siCtrl and siFTO for 72 hours, treated with 300 μM TBHP for 1 hour, and then 10 μM DCFDA for 30 min. The DCFDA levels were normalized to corresponding unstained control. (**D**) Intracellular ROS levels were determined by carboxy-DCFDA staining in 786-M1A cells transfected with siCtrl and siFTO for 72 hours, treated with 200 μM TBHP for 1 hour, and then 10 μM DCFDA for 30 min. The DCFDA intensities were normalized to corresponding unstained control. Each column represents the mean ± SD. Biological replicates (*n* = 3) were analyzed in each group. Statistically significant differences are indicated: *P < 0.05; **P < 0.01; ***P < 0.001; determined by the Student’s two-tailed *t* test.

In addition to being used for reductive carboxylation, glutamine is also used in the biosynthesis of glutathione (GSH), a key factor controlling intracellular ROS levels (*31*). Therefore, we next determined whether FTO inhibition affects intracellular GSH and ROS levels. Genetic inhibition of FTO decreased the total GSH levels and the ratio of reduced GSH to oxidized GSH (GSSG) within the UMRC2 and 786-M1A cells (Fig. 2, A and B). The decrease in GSH/GSSG ratios in FTO knockdown cells was associated with an increase in intracellular ROS as shown by increased 2′,7′-dichloroflourescin diacetate (DCFDA) staining in FTO knockdown cells at baseline and in combination with the ROS-inducing agent *tert*-butyl hydroperoxide (TBHP) (Fig. 2, C and D). Overall, these data indicate that FTO inhibition reduces GSH biosynthesis and increases ROS levels in ccRCC cells.

### FTO inhibition increases DNA damage and reduces the growth of ccRCC cells through the regulation of pyrimidine synthesis and ROS

**Fig. 3.**
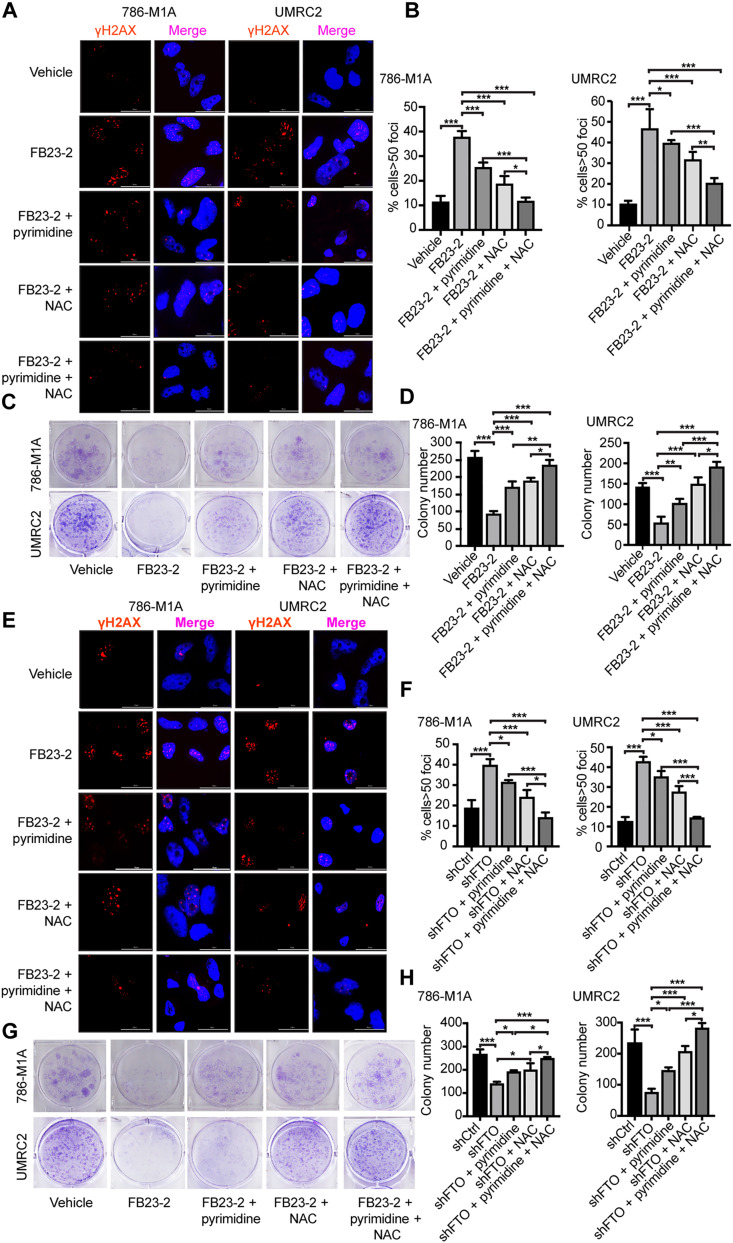
FTO inhibition increases DNA damage and impairs the growth of ccRCC cells, through decreased pyrimidine synthesis and increased ROS. (**A** and **B**) Cells were cultured in media with vehicle or 5 μM FB23-2 and supplemented with pyrimidines (cytidine 5 μM, thymidine 5 μM, and uridine 5 μM), NAC (4 mM), or a combination of pyrimidines and NAC for 72 hours. Immunofluorescence analysis of γH2AX foci in 786-M1A cells and UMRC2 cells (A). Bar graphs show the percentage of nuclei with >50 γH2AX foci (B). At least 50 nuclei for each biological replicate were analyzed. (**C** and **D**) Colony formation assays examined the growth and survival of 786-M1A and UMRC2 cells treated with FB23-2 (5 μM), FB23-2 (5 μM) + pyrimidines (5 μM), FB23-2 (5 μM) + NAC (4 mM), and FB23-2 (5 μM) + pyrimidines (5 μM) + NAC (4 mM). (**E** and **F**) 786-M1A and UMRC2 shCtrl and shFTO cells were pretreated with Dox (2 μg/ml) for 5 days and then the cells were supplemented with pyrimidines (5 μM), NAC (4 mM), or a combination of pyrimidines and NAC for 72 hours. Immunofluorescence analysis of γH2AX foci (E). Percentage of nuclei with greater than 50 γH2AX foci (F). At least 50 nuclei for each biological replicate were analyzed. (**G** and **H**) The 786-M1A and UMRC2 shCtrl and shFTO cells were pretreated with Dox (2 μg/ml) for 5 days, then with or without supplementation with 5 μM pyrimidines, 4 mM NAC, or the combination. Cell growth and survival were determined by 2D colony formation assays. Each column represents the mean ± SD. Biological replicates (*n* = 3) were analyzed in each group. Statistically significant differences are indicated: *P < 0.05; **P < 0.01; ***P < 0.001; determined by the Student’s two-tailed *t* test.

Our data above indicate that FTO inhibition results in decreased pyrimidine biosynthesis and increased intracellular ROS, which can lead to increased DNA damage. Therefore, we next investigated whether FTO inhibition increases DNA damage in ccRCC cells. The phosphorylation of the histone variant H2AX (γ-H2AX) is a marker of DNA damage (*32*). FTO inhibition with FB23-2 and genetic shRNA knockdown increased γ-H2AX foci formation within 786-M1A and UMRC2 cells (fig. S2, A to D). To determine whether FTO inhibition increased DNA damage in ccRCC cells through decreased pyrimidines and/or increased ROS, we treated the cells with FB23-2, pyrimidine nucleobases, the antioxidant *N*-acetylcysteine (NAC), or both pyrimidine nucleobases and NAC. Either pyrimidine nucleobase addition or NAC treatment partially rescued the increase in γ-H2AX foci formation in FB23-2–treated cells (Fig. 3, A and B). However, combining pyrimidine nucleobases and NAC produced an additive effect on reducing γ-H2AX foci formation (Fig. 3, A and B), indicating that FTO inhibition–induced DNA damage is mediated through reduced pyrimidine biosynthesis and increased ROS in ccRCC cells.

To determine the role of pyrimidines and ROS in FTO-mediated ccRCC growth and survival, we performed clonogenic assays in FB23-2–treated cells alone and in combination with pyrimidine nucleobases and NAC. FTO inhibition with FB23-2 reduced 786-M1A and UMRC2 growth and survival (Fig. 3, C and D). Treatment with pyrimidine nucleobases or NAC partially restored the growth and survival of FB23-2–treated cells, while the combination of pyrimidine nucleobases and NAC had an additive effect on ccRCC growth and survival (Fig. 3, C and D). Using shRNA-mediated FTO knockdown, we further confirmed that genetic inhibition of FTO promotes DNA damage and reduces ccRCC growth and survival through the regulation of pyrimidine biosynthesis and ROS. FTO knockdown cells showed increased levels of γ-H2AX foci formation that were rescued upon the addition of pyrimidine nucleobases and NAC (Fig. 3, E and F). Similarly, FTO knockdown reduced the growth and survival of 786-M1A and UMRC2 cells in a pyrimidine- and NAC-dependent manner (Fig. 3, G and H).

### FTO regulates SLC1A5 expression to promote ccRCC glutamine reductive carboxylation and GSH synthesis

**Fig. 4.**
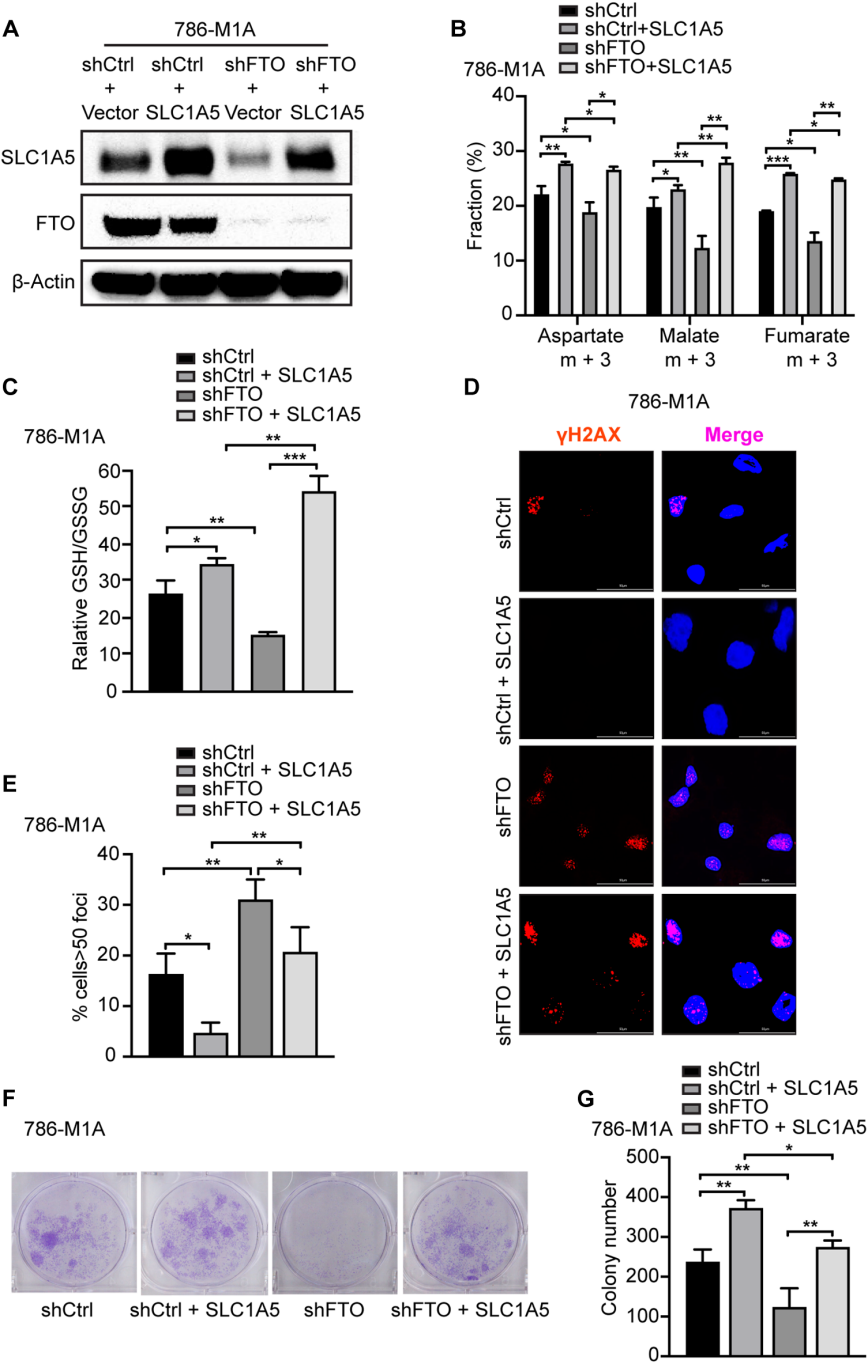
FTO regulates SLC1A5 to promote DNA damage and tumor growth. (**A**) Western blot analysis of FTO, SLC1A5, and B-actin in shCtrl, shCtrl + SLC1A5, shFTO, and shFTO + SLC1A5 786-M1A cells. 786-M1A shCtrl and shFTO cells were pretreated with Dox (2 μg/ml) for 5 days, then transfected with pcDNA-3.1 vector or pcDNA-3.1-SLC1A5 plasmid for 48 hours before extracting proteins. (**B**) U-13C glutamine tracing in cells. The fractions of m + 3 aspartate, malate, and fumarate were analyzed using LC-MS. (**C**) Relative GSH/GSSG ratio in shCtrl and shFTO 786-M1A cells pretreated with Dox (2 μg/ml) for 5 days, transfected with pcDNA-3.1 vector or pcDNA-3.1-SLC1A5 plasmid for 48 hours. (**D** and **E**) Immunofluorescence analysis of γH2AX foci of 786-M1A shCtrl and shFTO cells pretreated with Dox (2 μg/ml) for 5 days, then transfected with pcDNA-3.1 vector or pcDNA-3.1-SLC1A5 plasmid for 48 hours. Bar graphs show the percentage of nuclei with >50 γH2AX foci per nucleus. At least 50 nuclei for each biological replicate were analyzed. (**F** and **G**) Colony formation assays examined the growth and survival of shCtrl, shCtrl + SLC1A5, shFTO, and shFTO + SLC1A5 786-M1A cells. Macroscopic images of representative colonies formed in each group (F). Quantification of total colony number in each group (G). Each column represents the mean ± SD. Biological replicates (*n* = 3) were analyzed in each group. Statistically significant differences are indicated: *P < 0.05; **P < 0.01; ***P < 0.001; determined by the Student’s two-tailed *t* test.

We previously identified the glutamine transporter SLC1A5 as an m^6^A-regulated FTO target in ccRCC cells that promotes ccRCC growth and survival (*26*). SLC1A5 protein expression has previously been shown to correlate with reduced overall survival in patients with ccRCC (*33*). In an independent analysis of SLC1A5 expression in the ccRCC The Cancer Genome Atlas (TCGA) dataset, we confirmed that high SLC1A5 mRNA expression correlates with reduced survival in patients with ccRCC (fig. S3A). In addition, we demonstrate that SLC1A5 knockdown reduces glutamine uptake in ccRCC cells as shown by a reduction in the m + 5/m + 0 ratio in SLC1A5 knockdown cells (fig. S3, B and C). To determine whether SLC1A5 is a key downstream target of FTO promoting glutamine reprogramming, growth, and survival in ccRCC cells, we ectopically expressed SLC1A5 in FTO knockdown cells (Fig. 4A). U-^13^C glutamine tracing studies revealed that SLC1A5 expression was sufficient to restore the glutamine-derived reductive metabolites m + 3 aspartate, m + 3 malate, and m + 3 fumarate in FTO knockdown cells, indicating that SLC1A5 is an important FTO target driving glutamine-driven reductive carboxylation in ccRCC cells (Fig. 4B). Similarly, SLC1A5 expression in FTO knockdown cells increased the GSH/GSSG ratio and reduced DNA damage as determined by γH2AX foci formation (Fig. 4, C to E, and fig. S4, A to D). Last, we determined whether SLC1A5 is a key driver of FTO-mediated growth and survival in ccRCC cells. Restoration of SLC1A5 expression in FTO knockdown cells enhanced growth and survival (Fig. 4, F and G, and fig. S4, E and F). Overall, our data indicate that SLC1A5 is an important downstream FTO target driving glutamine reprogramming, growth, and survival of ccRCC cells.

### FTO inhibitors enhance the efficacy of PARP inhibitors

**Fig. 5.**
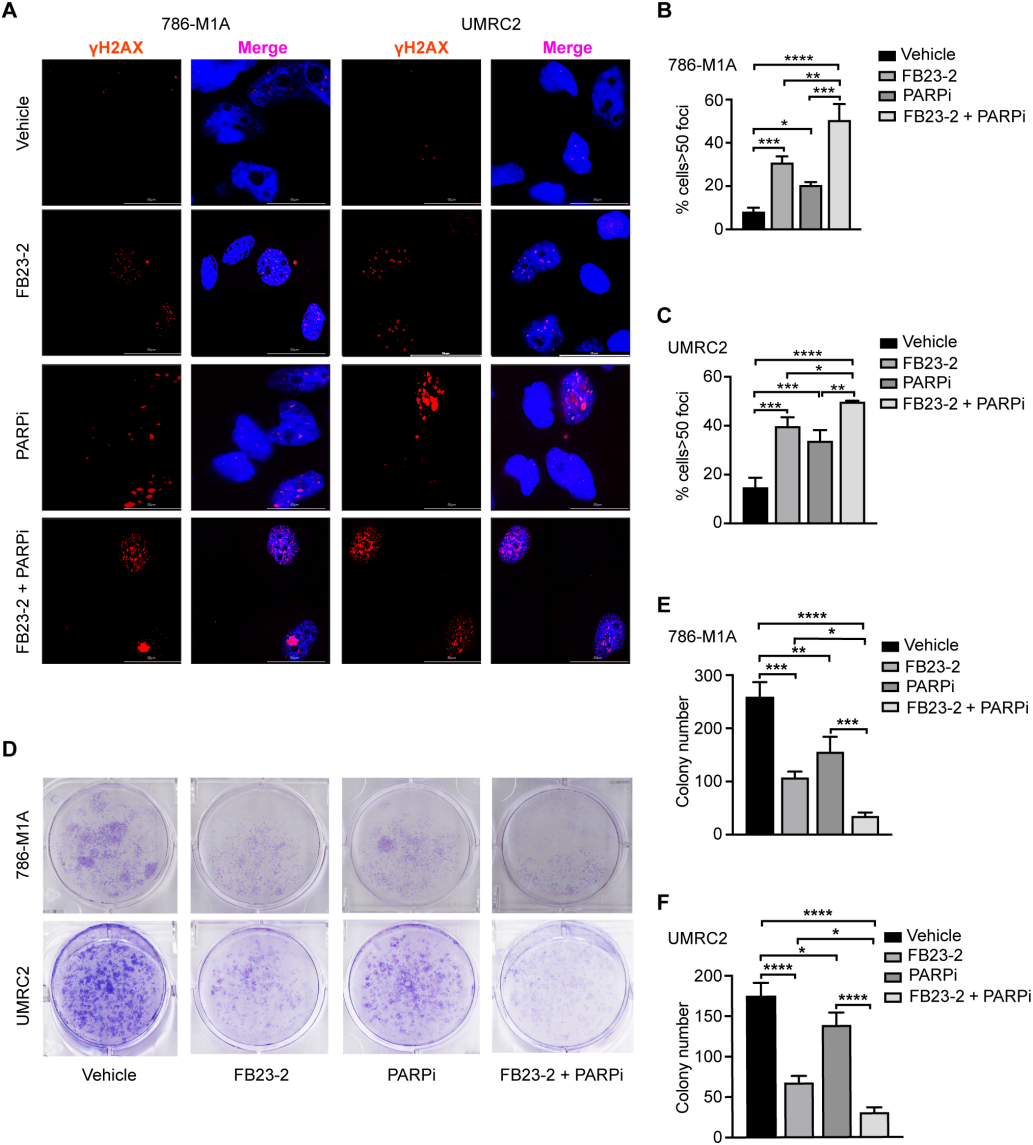
FTO inhibition enhances the efficacy of the PARP inhibitor talazoparib in ccRCC cells. (**A** to **C**) Immunofluorescence analysis of γH2AX foci of 786-M1A and UMRC2 cells pretreated with 5 μM FB23-2 and/or 1 μM PARPi (talazoparib) for 72 hours. Bar graphs show the percentage of nuclei with >50 γH2AX foci per nucleus. At least 50 cells for each biological replicate were analyzed. (**D** to **F**) 786-M1A and UMRC2 cells were treated with vehicle or 5 μM FB23-2 in the presence or absence of 1 μM PARPi (talazoparib) in colony formation assays. Each column represents the mean ± SD. Biological replicates (*n* = 3) were analyzed in each group. Statistically significant differences are indicated: *P < 0.05; **P < 0.01; ***P < 0.001, ****P < 0.0001; determined by the Student’s two-tailed *t* test.

**Fig. 6.**
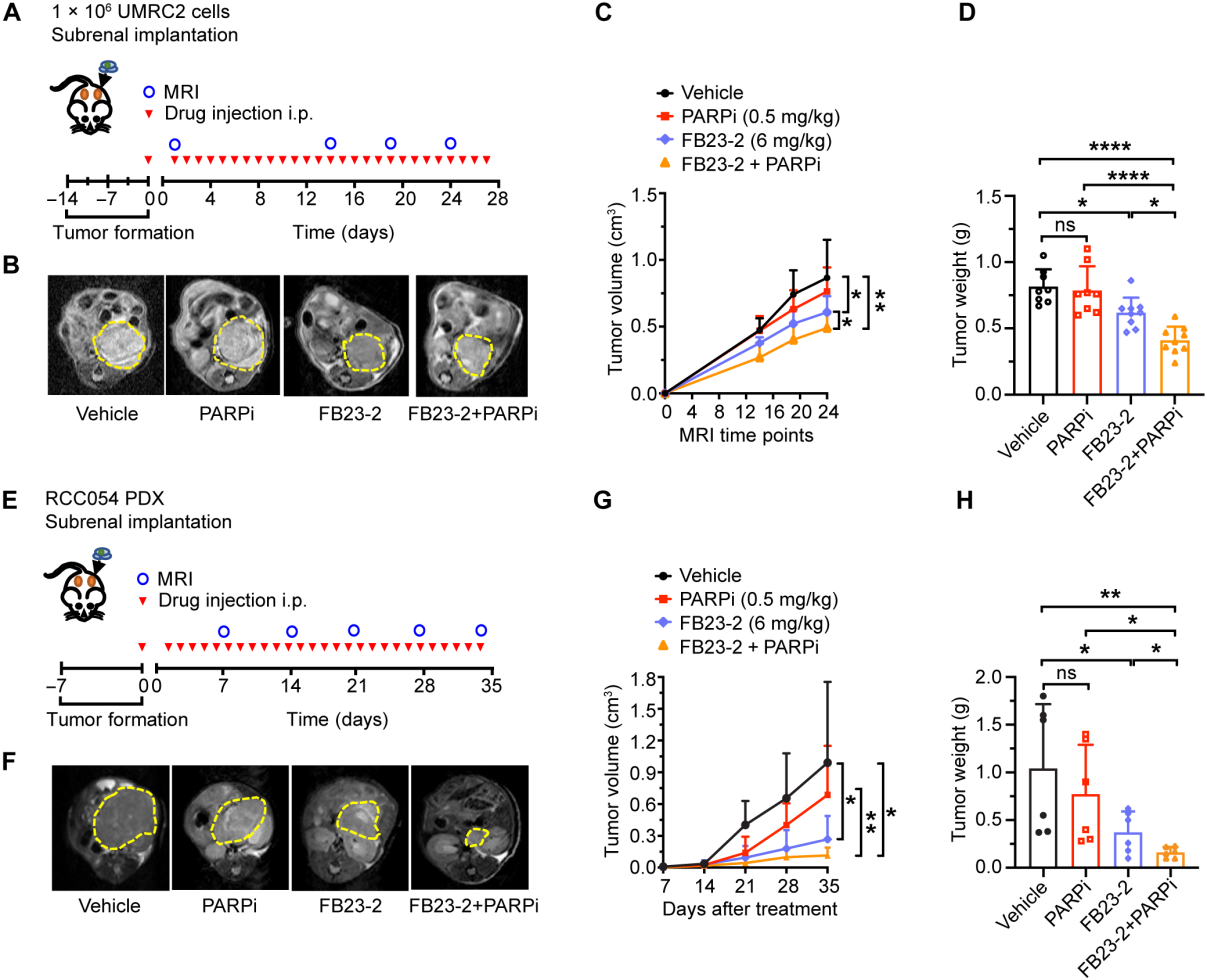
FTO inhibition enhances the efficacy of the PARP inhibitor talazoparib to suppress ccRCC tumor growth. (**A**) Schematic of the UMRC2 in vivo therapeutic study. Cells were implanted under the renal capsule (*n* = 8 to 9 per group). (**B**) Representative MRI images of kidneys from mice treated with of FB23-2 (6 mg/kg) or PARPi (0.5 mg/kg, talazoparib) or combination (FB23-2 with PARPi). Dashed yellow line outlines the tumor area. (**C**) Tumor volume was monitored via MRI. (**D**) Weight of tumors harvested at endpoint. (**E**) Schematic of the RCC054 PDX in vivo therapeutic study. Tissues were implanted under the renal capsule (*n* = 6 to 7 per group). (**F**) Representative MRI images of kidneys in mice treated with FB23-2 (6 mg/kg) or PARPi (talazoparib, 0.5 mg/kg) or combination (FB23-2 with PARPi). The dashed yellow line outlines the tumor area. (**G**) Tumor volume was monitored via MRI. (**H**) Weight of tumors harvested at endpoint (*n* = 6 per group). Each column represents the mean ± SD. Statistically significant differences are indicated: *P < 0.05; **P < 0.01; ****P < 0.0001; determined by the student’s two-tailed *t* test.

Our data above indicate that both genetic and pharmacologic inhibition of FTO reduces ccRCC growth and survival associated with increased DNA damage. Therefore, we sought to determine the therapeutic potential of targeting FTO alone and in combination with other agents that induce DNA damage or influence DNA repair. Preclinical studies in ccRCC models have shown that efficacy of GLS1 glutaminase inhibitors can be enhanced with poly(ADP-ribose) polymerase (PARP) inhibitors (PARPis) (*15*). Therefore, we investigated the efficacy of the FB23-2 alone and in combination with talazoparib, an FDA-approved PARPi (*34*). FTO inhibition with FB23-2 increased DNA damage in combination with talazoparib (Fig. 5, A to C). This increase in DNA damage in the combination treatment was associated with decreased ccRCC growth and survival in two-dimensional (2D) colony assays (Fig. 5, D to F). To determine the efficacy of FB23-2 alone and in combination with talazoparib in ccRCC tumor progression, we treated mice with established UMRC2 tumors under the renal capsule. FB23-2 (up to 20 mg/kg) has been previously used in preclinical models and found to be a safe and specific FTO inhibitor (*30*). We confirmed the safety of our FB23-2 treatment [6 mg/kg, intraperitoneally (i.p.) daily], both alone and in combination with talazoparib (0.5 mg/kg, i.p. daily), in mice bearing UMRC2 orthotopic tumors, with no observed differences in body weight among the treatment groups (fig. S5A). FB23-2 treatment (6 mg/kg, i.p. daily) reduced ccRCC tumor growth under the renal capsule compared to the vehicle treatment (Fig. 6, A to D). UMRC2 is resistant to talazoparib treatment alone at 0.5 mg/kg; however, the combination of FB23-2 with talazoparib reduced tumor growth compared to each single agent (Fig. 6, C and D, and fig. S5B). Next, we evaluated the efficacy of FB23-2 alone and in combination with talazoparib in a ccRCC patient–derived xenograft (PDX, RCC054) established from a VHL-deficient ccRCC colon metastasis (*35*). Single-agent FB23-2 as well as combination therapy with talazoparib reduced the growth of RCC054 under the renal capsule (Fig. 6, E to H, and fig. S5C). These findings demonstrate that FTO inhibition is sufficient to inhibit ccRCC tumor progression in cell line–derived xenografts and PDX and its effect on tumor growth can be enhanced with PARP inhibition.

## DISCUSSION

Metabolic reprogramming is a hallmark feature of ccRCC. Thus, identifying targetable factors that promote metabolic rewiring has the potential to improve outcomes in patients with ccRCC. The RNA demethylase enzyme FTO is emerging as an important therapeutic target with oncogenic activity in ccRCC. Here, we demonstrate that FTO is a key epitranscriptomic regulator of glutamine reprogramming in ccRCC. Genetic and pharmacologic inhibition of FTO reduced glutamine uptake, reductive carboxylation, and the GSH/GSSG ratio, leading to DNA damage and subsequently inhibiting ccRCC growth and survival. FTO inhibition alone and in combination with the PARPi talazoparib reduced the growth of cell line–derived xenografts and PDX. These findings identify FTO as a key epitranscriptomic regulator of glutamine metabolic reprogramming in ccRCC, suggesting that inhibiting FTO could be an effective therapeutic strategy for targeting glutamine reprogramming in ccRCC.

Our findings add to the emergence of FTO as an important epitranscriptomic regulator of cancer metabolic reprogramming. Recent studies have identified a role for FTO in the regulation of glycolysis. In acute myeloid leukemia cells, FTO promotes the expression of phosphofructokinase platelet and lactate dehydrogenase B expression, two key factors in aerobic glycolysis (*24*). In melanoma cells, FTO promotes glycolytic metabolism through the activation of c-Jun, JunB, and CCAAT/enhancer binding protein β (C/EBPB) (*25*). In this model, FTO inhibition reduced the glycolytic activity of the cancer cells to restore glucose availability in the tumor microenvironment and restore CD8^+^ T cell function (*25*). Our study identifies an important role for FTO in glutamine uptake and metabolic reprogramming of ccRCC cells. Whether there is a direct and/or secondary role for FTO in the regulation of glucose metabolism in VHL-deficient ccRCC cells remains to be explored. In addition, this study sets the stage for future work to determine whether FTO promotes glutamine uptake, and reprogramming in other glutamine-dependent cancers remains to be explored.

Our study identifies SLC1A5 as an important downstream FTO target that promotes ccRCC glutamine reprogramming, growth, and survival. SLC1A5 is the primary glutamine transporter in cancer cells and has an important role in maintaining the growth and survival of glutamine-dependent cancers (*36*–*38*). Consequently, SLC1A5 is emerging as an important therapeutic target for glutamine-dependent cancers (*39*). While high SLC1A5 expression is associated with tumor progression and reduced overall survival in patients with ccRCC, the functional role of SLC1A5 in the growth and survival of ccRCC cells remains largely unknown (*33*). Here, we define a mechanism by which FTO promotes SLC1A5 expression to enhance ccRCC growth and survival through the regulation of glutamine metabolism.

Our study identifies a role for FTO in controlling DNA damage within ccRCC cells. Our data indicate that decreased pyrimidine synthesis and increased ROS may contribute to the DNA damage in FTO knockdown cells as pyrimidine nucleobases and NAC decreased DNA damage in these cells. Previous studies have established that ccRCC cells use glutamine for pyrimidine and GSH biosynthesis (*15*). Glutaminase inhibitors have been used in preclinical studies to inhibit glutamine-derived pyrimidine biosynthesis in ccRCC cells resulting in DNA replication stress and growth arrest that could be further leveraged with PARPis (*15*). Our studies show that FTO inhibition is an alternative strategy to induce DNA damage in ccRCC cells by reducing pyrimidine biosynthesis and increasing ROS levels. However, we also cannot exclude the possibility that FTO inhibition may increase DNA damage through additional mechanisms. For example, a recent study found that FTO promotes the expression of DNA polymerase θ (POLQ), a key protein in the microhomology-mediated end joining (MMEJ) pathway involved in DNA repair (*40*).

Together, our findings indicate that therapeutic targeting of FTO may be an effective strategy to enhance the efficacy of DNA damaging agents for the treatment of ccRCC. We demonstrate that therapeutic inhibition of FTO is sufficient to reduce ccRCC tumor growth and enhance the efficacy of PARPi treatment in human ccRCC xenografts. Future studies will evaluate the efficacy of FTO inhibitors in combination with additional combination therapy approaches.

Although our findings offer mechanistic insights into the functional role of FTO in ccRCC and provide proof-of-concept data supporting the potential of FTO inhibitors in combination therapy for ccRCC treatment, there are several limitations to this study. First, we have concentrated on the intrinsic role of FTO within cancer cells, without addressing how FTO inhibition might affect the tumor immune microenvironment. Because glutamine metabolism can also influence immune cell function, ongoing studies are investigating the mechanisms and therapeutic potential of targeting FTO in immune-competent models of ccRCC. Second, while current FTO inhibitors, such as FB23-2, are valuable tools for studying FTO pharmacological inhibition, their clinical utility remains limited due to poor pharmacodynamics. As clinical FTO inhibitors are being developed for the treatment of obesity and cancer, our study provides preclinical rationale for further investigations into the efficacy and safety of these inhibitors for ccRCC treatment.

## MATERIALS AND METHODS

### Patient samples

All patients participating in this study gave written informed consent for the collection and research use of their biological materials. The use of these samples was approved by the Stanford University Institutional Review Board (IRB no. 34175) in accordance with established ethical standards, as outlined in the US Common Rule. Tissue from patient RCC054, a 44-year-old male who underwent surgery for the resection of metastatic ccRCC from the colon, was obtained under an IRB-approved protocol with informed consent.

### Cell lines and cell culture

The human ccRCC cell line UMRC2 was provided as a gift from O. Iliopoulos [Massachusetts General Hospital (*15*)]. The 786-M1A, a metastatic derivative of the 786-0 cell line was generously provided by J. Massague [Memorial Sloan Kettering (*41*)]. All cell lines were tested for viability, morphology, and screened for mycoplasma and viral contamination by Charles River Laboratories. The cells were cultured in Dulbecco’s modified Eagle’s medium (DMEM) supplemented with 10% fetal bovine serum (FBS), maintained at 37°C with 5% CO_2_.

### Drug treatments

The FTO inhibitor FB23-2 (Sigma-Aldrich, SML2694, CAS: 2243736–45-8) was used for the treatment of ccRCC UMRC2 and 786-M1A cells at a final concentration of 5 μM. A 5 mM stock solution of FB23-2 was prepared by dissolving the compound in 100% dimethyl sulfoxide (DMSO), followed by dilution in DMEM to achieve the desired working concentration, maintaining a final DMSO concentration of 0.1% as recommended for in vitro assays. NAC (Sigma-Aldrich, A7250, CAS: 616-91-1) was dissolved in distilled water to generate a 400 mM stock solution, which was further diluted in DMEM to a final concentration of 4 mM. This concentration was used in in vitro assays to assess intracellular ROS levels in UMRC2 and 786-M1A cells, following a 72-hour treatment. In addition, cytidine (Sigma-Aldrich, cat. no. C4654, CAS: 65-46-3), thymidine (Sigma-Aldrich, cat. no. T1895, CAS: 50-89-5), and uridine (Sigma-Aldrich, cat. no. U3003, CAS: 58-96-8) are pyrimidines. To prepare the pyrimidine solution, cytidine, thymidine, and uridine were dissolved in 100 ml of double-distilled water, incubated at 37°C for 30 min, then sterilized by passing through a 0.2-μm filter. The solution was stored at 4°C, with a final concentration in the medium of 5 μM. The PARPi talazoparib (10 mM solution in DMSO) was sourced from Targetmol (catalog no. T6253), with a final working concentration of 1 μM for in vitro experiments.

### Immunofluorescence staining

Cells were seeded onto a coverslip in a 24-well plate. Once the cells reached 70% confluency, the culture medium was removed from each well and the coverslip was washed 3 times with phosphate-buffered saline (PBS). Following three rinses with ice-cold PBS and fixation with 4% paraformaldehyde for 15 min at room temperature, the cells were washed again with ice-cold PBS for 5 min. Next, 0.3% Triton X-100 was added to each well and incubated for 10 min at room temperature, followed by another wash with ice-cold PBS for 5 min. To block nonspecific binding, the cells were incubated with 2% BSA for 30 min. After blocking, the primary rabbit anti-γH2AX antibody (Cell Signaling Technology, catalog no. 9718, 1:400) was incubated overnight at 4°C. After PBS washes, the cells were incubated for 1 hour at room temperature with a fluorophore-conjugated secondary antibody, Alexa Fluor 594–conjugated goat anti-rabbit (Life Technologies, A11012, 1:1000). Following PBS washes, the slides were mounted using VECTASHIELD with 4′,6-diamidino-2-phenylindole (DAPI; catalog no. 101098-044), and images were captured using a Leica Dmi8 laser confocal fluorescence microscope. At least five 60× images and 50 cells total from each sample were imaged and then the percentage of cells with >50 γH2AX foci was calculated (*n* = 3 biological replicates per group). The data shown represent three independent experiments.

### TCGA analysis of SLC1A5 expression in ccRCC

The ccRCC TCGA RNA sequencing data were obtained from https://software.broadinstitute.org/morpheus. SLC1A5 expression was assessed by comparing normal tissues, ccRCC tumors, and different tumor stages. For survival analysis, the median expression of SLC1A5 (10.5) was determined, and survival curves were compared between patients with SLC1A5 expression greater than or equal to 10.5 (*n* = 297) and those with expression lower than 10.5 (*n* = 237) using Kaplan-Meier analysis.

### Animal studies

All animal procedures and welfare were approved by the Institutional Animal Care and Use Committee (IACUC) at Stanford University, in compliance with both institutional policies and NIH guidelines (protocol 9984). Studies were conducted in accordance with ARRIVE guidelines. For the orthotopic ccRCC xenograft model, slices of RCC054 PDX tumors were implanted beneath the renal capsule of male Rag2^−/−^ IL2rg^−/−^ double knockout mice aged 6 to 12 weeks. Rag2^−/−^ IL2rg^−/−^ double knockout mice are bred in our animal facility. UMRC2 cells were mixed with a solidified collagen plug (BD Bioscience) (100 μl containing 1 × 10^6^ ccRCC cells) and implanted under the renal capsule. Specifically, cells were suspended in ice-cold neutralized collagen, and 100 μl of this mixture was placed in a 12-well plate. After incubating the plate at 37°C for 60 min to allow the collagen plug to solidify, it was surgically inserted beneath the renal capsule using fine-tipped forceps. The time schedule for tumor monitoring and collection is presented in the figures. For all treatments, mice were randomized into treatment groups. Total tumor weight was calculated by subtracting the weight of the tumor-free kidney from the weight of the tumor-bearing kidney. Tumor volume was measured using a 7-T Bruker MRI system (Bruker, Germany) by acquiring T_2_-weighted images. Tumor growth was monitored in a blinded manner by analyzing these images. MRI data were processed using OsiriX software, with regions of interest manually outlined on T_2_-weighted images to encompass the tumor. For treatment, talazoparib (Targetmol, catalog no. T6253, US) was prepared in 10% dimethylacetamide (DMAc)/PBS and administered intraperitoneally at a dosage of 0.5 mg/kg per day. FB23-2 (Sigma-Aldrich, catalog no. SML2694, US) was dissolved in a mixture of 5% DMSO, 30% PEG-300 (polyethylene glycol, molecular weight 300), 5% Tween 80, and 60% double distilled water (ddH_2_O), and administered via intraperitoneal injection at 6 mg/kg per day.

### Liquid chromatography–mass spectrometry analysis

For glutamine isotope tracing, UMRC2 and 786-M1A cells were treated with either DMSO or 5 μM FB23-2 for 72 hours. The culture medium was then replaced with DMEM/F-12 lacking glutamine (Gibco; 21331020) or supplemented with 4 mM U-^13^C glutamine (Cambridge Isotope Laboratories; CLM-1822-H), glucose, and 10% dialyzed FBS (Gibco; 26400044) for 10 min or 2 hours of incubation. Results are presented as mean ± SD from three independent biological replicates. In the metabolic tracing study, cells were first washed with cold PBS and then lysed on ice for 15 min in 80% Ultra LC-MS acetonitrile. The lysate was centrifuged at 20,000*g* for 10 min, and the resulting supernatant was analyzed using mass spectrometry. Liquid chromatography was performed using an Agilent 1290 Infinity LC system (Agilent, Santa Clara, US) coupled with a Q-TOF 6545 mass spectrometer (Agilent, Santa Clara, US). Compound separation was achieved via hydrophilic interaction chromatography using a bridged ethylene hybrid (BEH) amide column (100 × 2.1 mm i.d., 1.7 μm; Waters) at 35°C with a flow rate of 0.3 ml/min. Mobile phase A was composed of 25 mM ammonium acetate and 25 mM ammonium hydroxide in water, while mobile phase B consisted of acetonitrile. The gradient elution program was set as follows: from 0 to 1 min, 85% B; from 1 to 12 min, transitioning from 85% B to 65% B; from 12 to 12.2 min, transitioning from 65% B to 40% B; and from 12.2 to 15 min, holding at 40% B. After this gradient, the column was re-equilibrated at 85% B for 5 min. The total runtime for the analysis was 20 min, with an injection volume of 5 μl. The Agilent Q-TOF was operated in negative ion mode with the following settings: ion spray voltage at 3500 V, nozzle voltage at 1000 V, fragmentor voltage at 125 V, drying gas flow at 11 liters/min, capillary temperature at 325°C, drying gas temperature at 350°C, and nebulizer pressure at 40 psi. The full scan range was established from 50 to 1600 *m*/*z* (mass/charge ratio), using reference masses of 119.0363 and 980.0164. The acquisition rate was set to 2 spectra per second. Targeted analysis, isotopologue extraction, and natural isotope abundance correction were conducted using Agilent Profinder B.10.00 (Agilent Technologies).

### 2D colony formation assays

Cell growth and survival were assessed through a 2D colony formation assay, following a previously described protocol. For monolayer colony formation analysis, 1000 viable cells (786-M1A and UMRC2) were seeded in each well of a six-well plate and cultured in DMEM 10% FBS medium supplemented with Hepes for 2 weeks. Three wells were assigned to each treatment group. After incubation, colonies were fixed with methanol and stained using 1% crystal violet dissolved in 100% ethanol. The colonies were then air dried, counted, and photographed.

For experiments involving genetic knockdown cells, 786-M1A and UMRC2 shCtrl and shFTO cells were pretreated with doxycycline (Dox, 2 μg/ml) for 5 days. For rescue experiments, shFTO cells were transfected with SLC1A5 plasmids using Lipofectamine 3000 (Thermo Fisher Scientific) according to the manufacturer′s instructions. After 72 hours post-transfection, cells from each group (shCtrl, shCtrl + SLC1A5, shFTO, and shFTO + SLC1A5) were trypsinized, counted, and seeded at a density of 1000 cells per well in six-well plates in complete medium with Hepes. Cells were cultured for 14 days, with the medium replaced every 2 to 3 days, until visible colonies formed. Colonies were subsequently fixed and stained with 1% crystal violet in 100% ethanol.

In drug treatment experiments, 1000 cells were plated in six-well plates incubated in 10% FBS DMEM medium with Hepes. After 24 hours, cells were treated with either vehicle, 5 μM FB23-2 (Sigma-Aldrich, catalog no. SML2694), 1 μM PARPi (talazoparib, Targetmol, catalog no. T6253), 5 μM pyrimidines (Cytidine, Sigma-Aldrich, catalog no. C4654; thymidine, Sigma-Aldrich, catalog no. T1895; uridine, Sigma-Aldrich, catalog no. U3003), or 4 mM NAC (Sigma-Aldrich, catalog no. A7250). Drug treatments were carried out for 14 days, with media refreshed after 7 days. All assays were conducted in triplicate.

### Establishment of stable FTO knockdown cell lines and siRNA

Dox-inducible human shFTO plasmids and Dox-inducible non-targeting shCtrl plasmids were acquired from Dharmacon (VSC11653 and V3SH11252-227). The stable cell lines of 786-M1A and UMRC2 shCtrl and shFTO are pretreated with Dox (2 μg/ml) for 5 days, with Dox-inducible shFTO knockdown efficiency verified by Western blot assay in 786-M1A and UMRC2 cells.

The ON-TARGETplus smart pool siRNAs for siCtrl (D-001810-10-20), siFTO (L-004159-01), and siSLC1A5 (L-007429-00-0005) were obtained from Dharmacon. Cells (1000 cells per well for 96-well plates; 50,000 cells per well for 12-well plates; 100,000 cells per well for 6-well plates) were seeded. Subsequently, the cells were transfected with a mixture of Lipofectamine RNAiMAX (13778, Thermo Fisher Scientific) and the corresponding siRNA(s) in conjunction with Opti-MEM media (Invitrogen), following the manufacturer’s protocol. After 48 hours post-transfection, the cells were trypsinized, washed, and replated for subsequent assays as detailed below.

### Western blot and antibodies

Total cell lysates were isolated in radioimmunoprecipitation assay buffer (RIPA) and proteins were quantified using the Pierce BCA Protein Assay Kit (Thermo Fisher Scientific). Protein samples were separated by gel electrophoresis on 4 to 12% bis-tris gels (Thermo Fisher Scientific) and transferred to a polyvinylidene difluoride membrane. Following blocking with 5% milk in phosphate-buffered saline with Tween 20 (PBST) buffer, the membranes were incubated overnight with primary antibodies at 4°C. The blots were developed using a chemiluminescence kit (Thermo Fisher Scientific, 34096) and visualized with the ChemiDoc XRS+ imaging system equipped with Image Lab Software (Bio-Rad Laboratories). The primary antibodies used were anti-FTO (1:1000, D2V1I, Cell Signaling Technology), anti-SLC1A5 (1:1000, ab237704, Abcam), and anti–β-actin (1:5000, 8H10D10, Cell Signaling Technology).

### GSH/GSSG ratio and intracellular ROS measurement

GSH/GSSG ratio was determined using GSH/GSSG-Glo (Promega, catalog V6611) according to the manufacturer’s protocol. The relative GSH/GSSG ratio was measured using a kit in UMRC2 and 786-M1A cells treated with DMSO or 5 μM FTO inhibitor (FB23-2) for 72 hours, as well as in Dox-inducible UMRC2 and 786-M1A shCtrl and shFTO cell lines over 72 hours. Intracellular ROS levels were assessed via carboxy-DCFDA staining in cells transfected with siCtrl or siFTO for 72 hours. For 786-M1A cells, treatment with 200 μM TBHP for 1 hour was followed by the addition of 10 μM DCFDA for 30 min. For UMRC2 cells, treatment with 300 μM TBHP for 1 hour was followed by the addition of 10 μM DCFDA for 30 min. DCFDA fluorescence intensities were normalized to corresponding unstained controls.

ROS levels in UMRC2 and 786-M1A cells were determined by staining of the cells with carboxy-DCFDA (Invitrogen, catalog C369). A concentrated stock solution 10 mM was prepared in high-quality DMSO. Once prepared, the stock solutions were used within a short time. Cells were washed with PBS and incubated with 10 μM carboxy-DCFDA for 10 min at 37°C. The cells were processed and analyzed using a BD LSRII Flow Cytometer System (BD Biosciences) and FlowJo software (Tree Star).

### Cell growth curves

UMRC2 and 786-M1A kidney cancer cells were seeded in black 96-well plates (Corning, catalog no. 3603) and cultured in DMEM media supplemented with 10% dialyzed FBS (Gibco, catalog no. A3382001) with or without 4 mM glutamine. At 24, 48, and 72 hours, Hoechst 33342 (20 mM, Thermo Fisher Scientific, catalog no. 62249) was added to a final concentration of 2 μM, followed by a 15-min incubation in the dark. Quantification of cell number was performed using a BioTek Cytation 5 imaging system (Agilent) in combination with Gen5 software, according to the manufacturer’s instructions.

### Caspase-3/7 activity assay

Caspase-3/7 activity in ccRCC cells was measured using the Apo-ONE Homogeneous Caspase-3/7 Assay kit (Promega, catalog no. G7790) according to the manufacturer’s instructions. UMRC2 and 786-M1A cells were seeded in black, clear-bottom 96-well plates (Corning, catalog no. 3603) at a density of 5000 cells per well in 100 μl of complete medium. The next day, cells were treated with or without 4 mM glutamine in DMEM supplemented with 10% dialyzed FBS for the indicated time points (24, 48, and 72 hours) and then 100 μl of Apo-ONE Caspase-3/7 reagent. The plate was incubated at room temperature for 1 hour. Fluorescence intensity was measured using the Cytation 5 imaging reader (Agilent Technologies) and quantified with Gen5 software (Agilent Technologies). Caspase-3/7 activity was normalized to background signal from wells containing medium and reagent only.

### Statistical analysis

Each experiment was repeated at least three times. Statistical significance was assessed by comparing mean values (±SD) using an analysis of variance (ANOVA) and Student’s *t* test for independent groups and was assumed for *P < 0.05; **P < 0.01; ***P < 0.001; **** *P* < 0.0001. All statistical analysis was performed using GraphPad Prism v10. Sample sizes were determined based on our previous data. No data were excluded from our analyses.
