# Supplementary material for: The RNA demethylase FTO promotes glutamine metabolism in clear cell renal cell carcinoma through the regulation of SLC1A5
Source: Sci Adv. 2025 Jun 18;11(25):eadv2417. doi: 10.1126/sciadv.adv2417 (PMC12175902; doi:10.1126/sciadv.adv2417)
Supplement: Supplementary file 1 — Figs. S1 to S5 [file sciadv.adv2417_sm.pdf]

Science Advances
AAAS

Supplementary Materials for

The RNA demethylase FTO promotes glutamine metabolism in clear cell renal cell carcinoma through the regulation of SLC1A5

Man Zhao *et al.*

Corresponding author: Erinn B. Rankin, erankin@stanford.edu

Sci. Adv. **11**, eadv2417 (2025)
DOI: 10.1126/sciadv.adv2417

This PDF file includes:

Figs. S1 to S5

Supplementary Figures

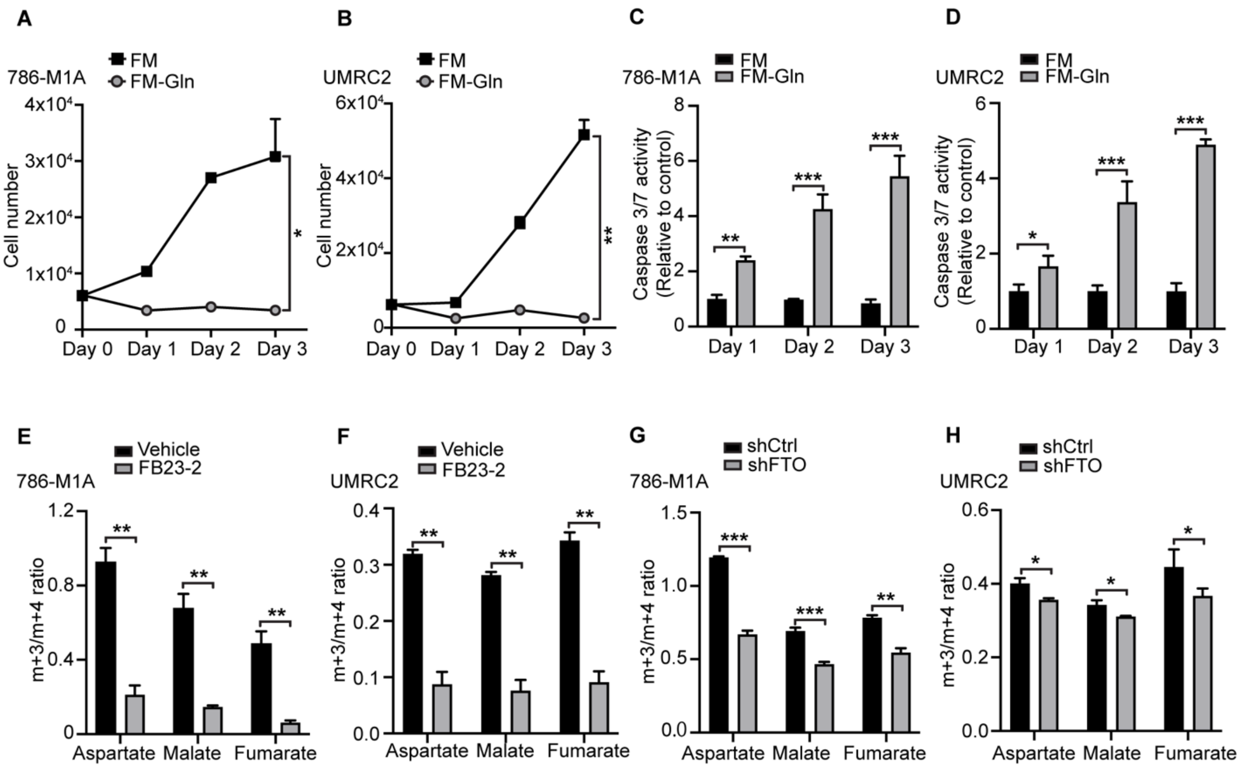

Fig. S1 Clear cell renal cell carcinoma (ccRCC) cells are glutamine dependent and FTO inhibition reduces glutamine-derived metabolism in ccRCC cells, related to Fig.1. (**A** and **B**) Growth curves of 786-M1A and UMRC2 cells cultured in the presence or absence of glutamine. Cells were cultured in either full medium (FM), consisting of glutamine-free DMEM supplemented with 10% dialyzed FBS and 4 mM glutamine, or in glutamine-deficient medium (FM-Gln), containing glutamine-free DMEM and 10% dialyzed FBS. (**C** and **D**) Caspase-3/7 activity in 786-M1A and UMRC2 cells after culture in the media conditions described above. At the end of the treatment period, caspase-3/7 activity was quantified using the Apo-ONE® Homogeneous Caspase-3/7 Assay according to the manufacturer′s protocol. (**E** and **F**) 786-M1A and UMRC2 cells were pretreated with DMSO or 5 μM FB23-2 for 72 hours, then labeled with U-^{13}C glutamine for 2 hours. The ratio of m+3/m+4 aspartate, m+3/m+4 malate and m+3/m+4 fumarate were analyzed using LC-MS. (**G** and **H**) shCtrl and shFTO of 786-M1A and UMRC2 cells were pretreated with doxycycline (Dox, 2 μg/mL) for 5 days, then labeled with U-^{13}C glutamine for 2 hours. The m+3/m+4 aspartate, malate and fumarate ratios were analyzed using LC-MS. Each column represents the mean ± SD. Biological replicates (n=3) were analyzed in each group. Statistically significant differences are indicated: *P<0.05; **P<0.01; ***P<0.001; determined by the student's two-tailed t test.

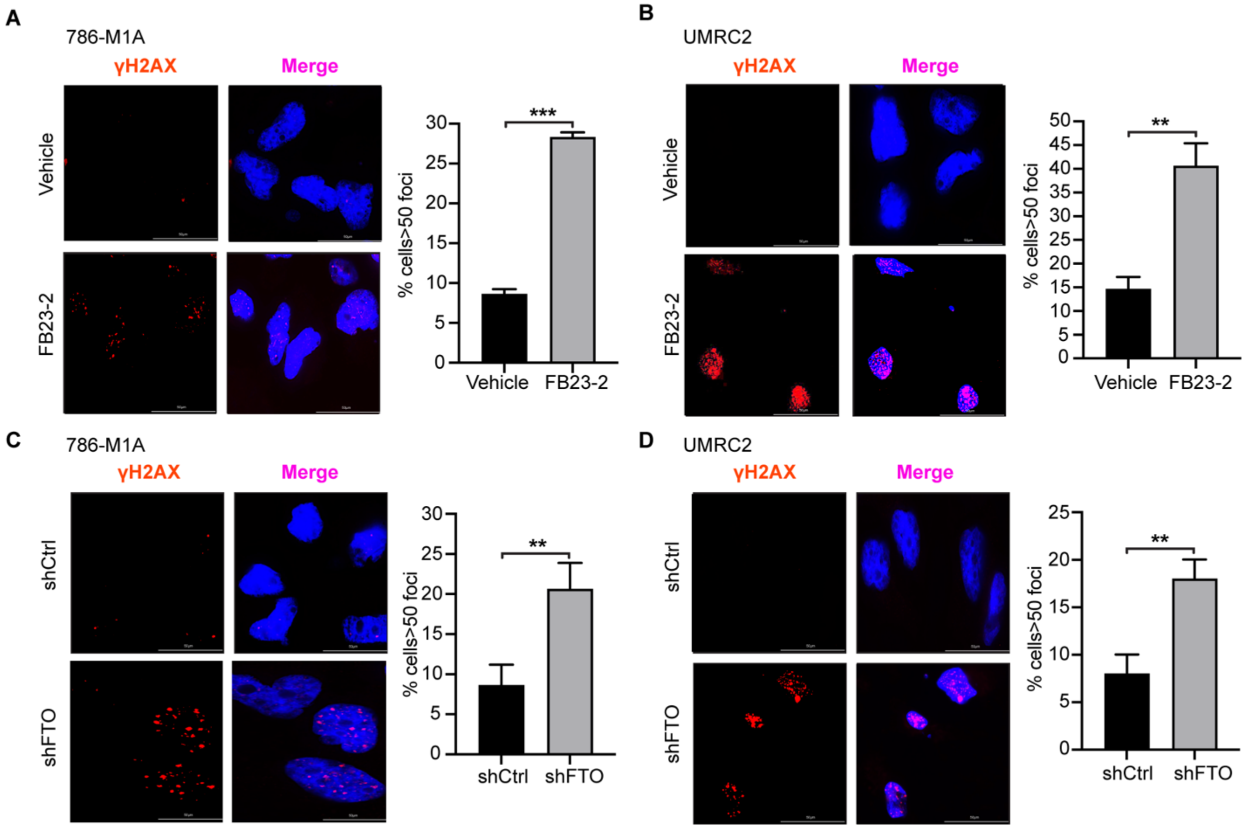

Fig. S2. FTO inhibition increases DNA damage in ccRCC cells, related to Fig.3. (**A-D**) Immunofluorescence analysis of γH2AX foci (red) in 786-M1A and UMRC2 cells treated with vehicle or 5 μM FB23-2 (A and B) and of 786-M1A and UMRC2 shCtrl and shFTO cells induced with doxycycline (Dox, 2 μg/mL) for 5 days (C and D). Bar graphs show the percentage of cells containing >50 γH2AX foci per nucleus. A minimum of 50 cells were analyzed for each biological replicate. Each column represents the mean ± SD. Biological replicates (n=3) were analyzed in each group. Statistically significant differences are indicated: **P*<0.05; ***P*<0.01; ****P*<0.001; determined by the student's two-tailed *t* test.

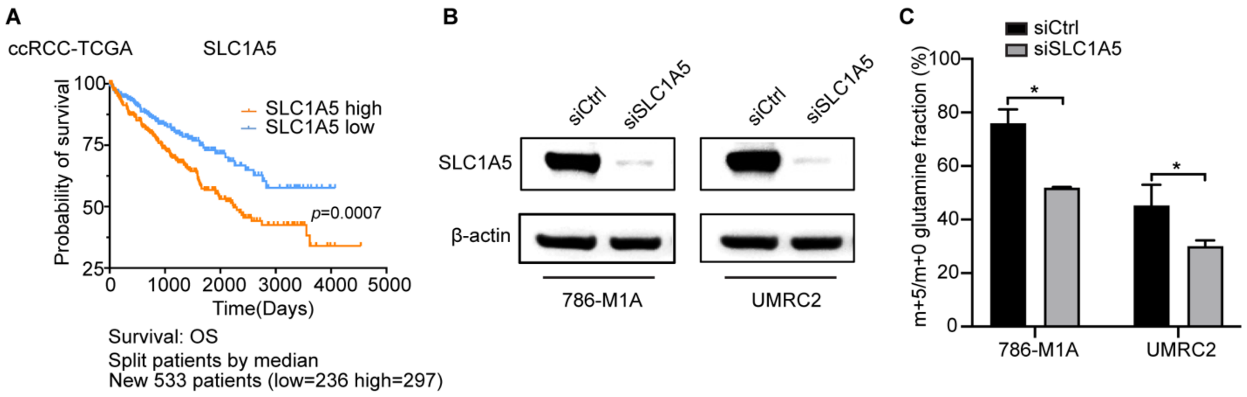

Fig. S3. SLC1A5 correlates with poor survival and promotes glutamine uptake in ccRCC cells, related to Fig.4. (**A**) Kaplan-Meier analysis of overall survival in ccRCC patients (ccRCC TCGA) with low and high SLC1A5 mRNA expression (n= 236 and 297 respectively, p=0.0007 with Log-rank (Mantel-Cox) test. (**B**) siSLC1A5 knockdown efficiency was verified by western blot analysis in 786-M1A and UMRC2 cells after 72 hours post-transfection. (**C**) 786-M1A and UMRC2 cells were pre-transfected with siSLC1A5 for 72 hours, then labeled with U-^{13}C glutamine for 2 hours. The ratio of m+5/m+0 glutamine was analyzed using LC-MS. Each column represents the mean ± SD. Biological replicates (n=3) were analyzed in each group. Statistically significant differences are indicated: *P<0.05 determined by the student's two-tailed t test.

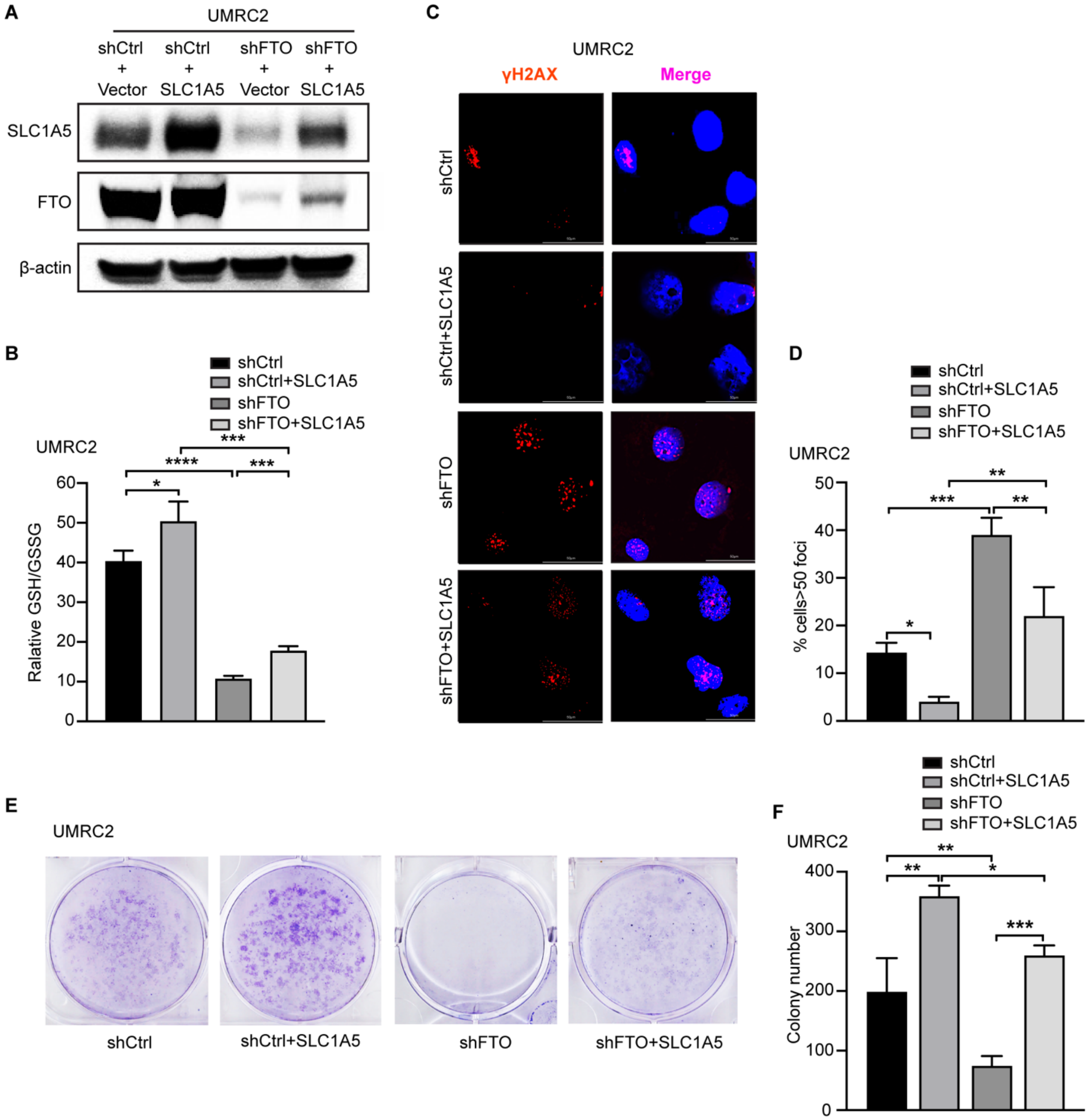

Fig. S4. FTO regulates SLC1A5 to promote DNA damage and ccRCC cell growth and survival, related to Fig.4. (**A**) Western blot analysis of FTO and SLC1A5 protein levels in shCtrl and shFTO UMRC2 cells pretreated with doxycycline (Dox, 2 μg/mL) for 5 days and transfected with pcDNA-3.1 vector or pcDNA-3.1-SLC1A5 plasmid for 48 hours. (**B**) Relative GSH/GSSG ratio in shCtrl, shCtrl+SLC1A5, shFTO, shFTO+SLC1A5 UMRC2 cells. (**C** and **D**) Immunofluorescence analysis of γH2AX foci in shCtrl and shFTO UMRC2 cells pretreated with doxycycline (Dox, 2 μg/mL) for 5 days and transfected with pcDNA-3.1 vector or pcDNA-3.1-SLC1A5 plasmid for 48 hours. Bar graphs show the percentage of cells with the >50 γH2AX foci per nucleus. At least 50 cells for each condition were analyzed. (**E** and **F**) Colony formation assays examined the growth and survival of UMRC2 cells described above. Each column represents the mean ± SD. Biological replicates (n=3) were analyzed in each group. Statistically significant differences are indicated: **P*<0.05; ***P*<0.01; ****P*<0.001; determined by the student's two-tailed *t* test.

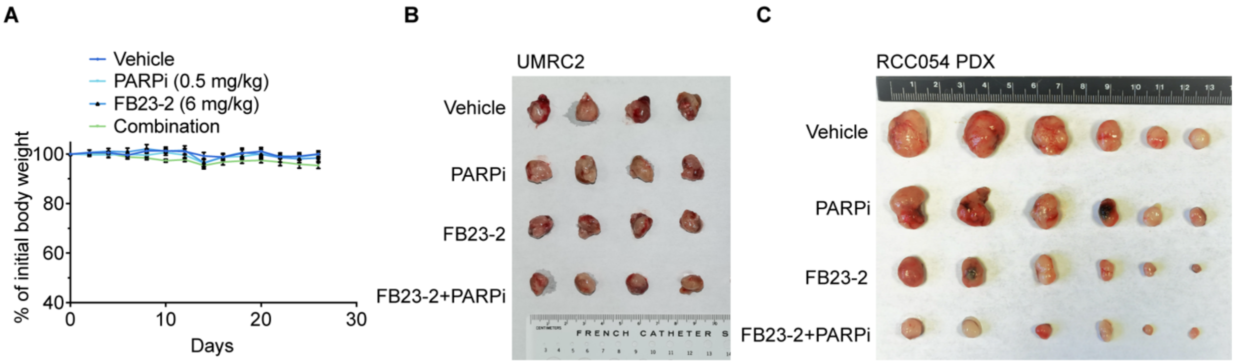

Fig. S5. FTO inhibition enhances the efficacy of PARP inhibitor to suppress ccRCC tumor growth, related to Fig. 6. (A) Body weights over time of the mice bearing orthotopic UMRC2 tumors treated with vehicle, PARPi (talazoparib, 0.5 mg/kg), FB23-2 (6 mg/kg) or the combination of FB23-2 with PARPi. (**B** and **C**) Macroscopic images of tumors harvested at endpoint after treatment of mice with vehicle, FB23-2 (6 mg/kg) or PARPi (talazoparib, 0.5 mg/kg) or the combination FB23-2 with PARPi.
